# Elevated Expression of the Integrin-Associated Protein PINCH Suppresses the Defects of *Drosophila melanogaster* Muscle Hypercontraction Mutants

**DOI:** 10.1371/journal.pgen.1003406

**Published:** 2013-03-28

**Authors:** Stephen M. Pronovost, Mary C. Beckerle, Julie L. Kadrmas

**Affiliations:** Huntsman Cancer Institute, Departments of Biology and Oncological Sciences, University of Utah, Salt Lake City, Utah, United States of America; McGill Universiy, Canada

## Abstract

A variety of human diseases arise from mutations that alter muscle contraction. Evolutionary conservation allows genetic studies in *Drosophila melanogaster* to be used to better understand these myopathies and suggest novel therapeutic strategies. Integrin-mediated adhesion is required to support muscle structure and function, and expression of Integrin adhesive complex (IAC) proteins is modulated to adapt to varying levels of mechanical stress within muscle. Mutations in *flapwing* (*flw*), a catalytic subunit of myosin phosphatase, result in non-muscle myosin hyperphosphorylation, as well as muscle hypercontraction, defects in size, motility, muscle attachment, and subsequent larval and pupal lethality. We find that moderately elevated expression of the IAC protein PINCH significantly rescues *flw* phenotypes. Rescue requires PINCH be bound to its partners, Integrin-linked kinase and Ras suppressor 1. Rescue is not achieved through dephosphorylation of non-muscle myosin, suggesting a mechanism in which elevated PINCH expression strengthens integrin adhesion. In support of this, elevated expression of PINCH rescues an independent muscle hypercontraction mutant in muscle myosin heavy chain, *Mhc^Samba1^*. By testing a panel of IAC proteins, we show specificity for PINCH expression in the rescue of hypercontraction mutants. These data are consistent with a model in which PINCH is present in limiting quantities within IACs, with increasing PINCH expression reinforcing existing adhesions or allowing for the *de novo* assembly of new adhesion complexes. Moreover, in myopathies that exhibit hypercontraction, strategic PINCH expression may have therapeutic potential in preserving muscle structure and function.

## Author Summary

A wide variety of diseases of the muscle are caused by mutations that alter either the actin and myosin contractile machinery or its regulation. One class of mutations of interest results in hypercontraction of the muscle—actin and myosin fibers contract, but cannot efficiently relax. We have used the fruit fly as a model to study these mutations because of the striking similarity of fly and human muscle and because of the many genetic techniques that are available in the fly. Using a genetic approach we identified a protein, PINCH, whose increased expression can rescue the defects observed in hypercontraction mutants. PINCH is a component of integrin adhesion complexes, responsible for anchoring cells in their environment. This suggests that strengthening the anchorage of muscles via PINCH may be an effective strategy to prevent or reduce the muscle damage that occurs in diseases of muscle hypercontraction.

## Introduction

Numerous human diseases, including muscular dystrophies [1], [2] and cardiomyopathies [3], [4], result from mutations that alter muscle contraction. The evolutionary conservation and genetic tractability of *Drosophila melanogaster* have made it an attractive system in which to characterize these genes and mutations [5], [6]. A collection of mutants in *Drosophila* exhibits myopathy due to muscle hypercontraction, leading to a range of phenotypes from flightless adults to early lethality [7]–[12]. Upon hypercontraction, these mutants can compensate in a variety of ways. Through a genetic response, specific mutations in genes such as the *Myosin heavy chain* (*Mhc*) can decrease overall actomyosin force, which is sufficient to suppress hypercontraction defects [11], [13]. Hypercontraction can also induce compensatory changes in gene expression. For instance, expression profiling of *Mhc^Samba^* hypercontraction mutants suggested an extensive actin cytoskeletal remodeling response, as well as upregulation of non-muscle myosin phosphatase expression to promote relaxation of the actomyosin cytoskeleton [14]. Notably, upregulation of genes for integrin adhesive complex (IAC) proteins like Paxillin and Talin was observed as well [14]. This suggests that increasing the expression levels of IAC proteins can strengthen integrin-based adhesions at muscle termini to cope with the increased mechanical stress of muscle hypercontraction. Integrin-mediated adhesion has been shown to help maintain muscle cytoarchitecture and sarcomeric integrity [15], and mechanical force regulates the rate of integrin turnover [16]. Consistent with a key role for IAC gene expression in responding to muscle contraction, genes including UNC-97/PINCH show decreased mRNA levels in *C. elegans* muscles developed in the microgravity of spaceflight [17]. Furthermore, review of the ground controls (deposited in NCBI's Gene Expression Omnibus: http://www.ncbi.nlm.nih.gov/geo/query/acc.cgi?acc=GSE36358) also reveals increased UNC-97 mRNA levels under conditions of hypergravity. This supports the idea that modulating gene expression of IAC components may be an evolutionarily conserved mechanism to adapt to widely divergent levels of mechanical stress to maintain muscle integrity.

Flapwing (Flw) is a serine/threonine Protein Phosphatase 1β (PP1β) that acts broadly upon many phosphorylated protein substrates, but it is required solely for its activity in dephosphorylating non-muscle myosin regulatory light chain (MRLC), encoded by *spaghetti squash* (*sqh*) in *Drosophila* [11]. Phosphorylation of Sqh causes an activating conformational change that promotes contraction of the actomyosin cytoskeleton. Sqh dephosphorylation is a key step in actomyosin relaxation. Thus strong loss-of-function mutants in *flw* exhibit hyperphosphorylation of non-muscle myosin. Notably, though *flw* does not genetically interact with the muscle version of MRLC (*Mlc2*) [10], the main defect observed in *flw* mutants is hypercontraction of myofibrillar myosin, eventually resulting in detachment of striated muscles and subsequent lethality at both larval and pupal stages [10]. Though not directly responsible for the generation of contractile force, non-muscle myosin is necessary for the proper development of myofibrils [18]. Hyperphosphorylation of Sqh disrupts actin cytoskeletal dynamics in many cell types, but the consequences appear greatest in contractile muscle tissue, both in larval body wall muscle and in adult indirect flight muscle [10]. The molecular connection between hypercontracted cytoskeleton and hypercontraction and detachment of muscle myofibrils is indirect, and currently not well defined.

We hypothesized that the *flw* muscle hypercontraction phenotype could be rescued by strengthening the anchoring of muscles at integrin-based adhesion sites to better withstand the force of hypercontraction. We show here that moderately elevated expression of the IAC protein PINCH alleviates the phenotypes of *flw* mutants. PINCH is an integrin-associated LIM domain adaptor protein encoded by the *steamer duck* (*stck*) gene in *Drosophila*. PINCH is part of an evolutionarily conserved, high affinity protein complex comprised of Ras Suppressor 1 (RSU1), Integrin-linked kinase (ILK) and Parvin that is targeted to the cell membrane at sites of integrin adhesion [19]–[22]. This complex of proteins has a critical role in adhesion maintenance, particularly in muscle attachment. In *Drosophila*, null mutants in PINCH, ILK, and Parvin each die late in embryogenesis as a consequence of failure of integrin-based muscle attachment sites within the segmental musculature [23]–[25]. Null mutations in RSU1 show milder defects in integrin adhesion between the epithelial layers of the wing [19]. Notably, mammalian α-Parvin and ILK have been shown to be negative regulators of contractility in certain cellular contexts [26], [27]. The RSU1, PINCH, ILK and Parvin proteins have been shown to mutually stabilize each other [19], [22], [28]–[30], suggesting that the complex as a whole might help delay the onset or reduce the extent of the damage exhibited in hypercontraction mutants by stabilizing adhesions. Data presented here support this idea. We show that PINCH must be capable of directly binding both ILK and RSU1 in order to suppress the defects of the *flw* mutant.

We demonstrate that structural stabilization of integrin-based adhesions by PINCH is likely to be a mechanism for the rescue of *flw* mutants. PINCH does not appear to alleviate hypercontraction of the myofibrils, as muscle attachment is better maintained even in the presence of muscle hypercontraction. We also eliminate phospho-regulation as a plausible mechanism, as elevated PINCH expression does not alter the phosphorylation status of the only essential Flapwing substrate, MRLC/Sqh. We show that moderately elevated expression of PINCH partially rescues another hypercontraction mutant, the *Samba^1^* allele of *Mhc*. Furthermore, we show that among a panel of IAC proteins tested, elevated expression of Talin also affords marginal rescue of *Samba^1^* hypercontraction defects. However, transgenic Talin expression is unable to afford rescue of *flw* larval lethality, underscoring the specificity of PINCH expression in strengthening adhesion under multiple conditions of hypercontraction. These studies have broad implications for understanding and treating a variety of myopathies and muscle degenerative diseases.

## Results

**Figure 1:**
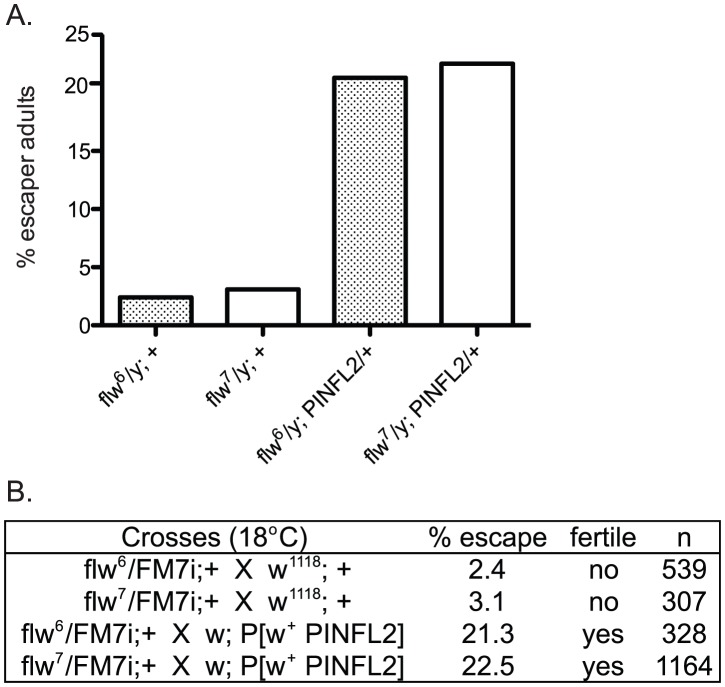
Expression of transgenic PINCH increases the number of *flw* adult male escapers and restores their fertility. A.) Bars show the frequency of escapers of the indicated genotypes. B.) The genetic crosses employed are shown, as well as the fertility status of the escapers. n is the total number of progeny of all genotypes resulting from the cross.

In a recent report, an interaction between mammalian Protein Phosphatase-1 (PP1) and the integrin-associated protein PINCH was described [31]. Because of the similar muscle detachment phenotypes exhibited by loss-of-function mutants for both PINCH [23] and PP1β [10] in the fly, we set out to test whether the *Drosophila* proteins also interact. Our analyses included two recessive alleles of the PP1β family member, *flapwing. flw^6^* is a point mutant with decreased substrate affinity, and *flw^7^* contains a *lacZ* enhancer trap inserted in the *flw* 5′ UTR which reduces the level of Flw expression [10]. Both of these mutants are strong loss-of-function alleles, but do not entirely eliminate Flapwing phosphatase activity or protein expression in male hemizygotes. In a wild type PINCH background, we combined a wild type PINCH-Flag transgene expressed from the native PINCH promoter with each of these *flw* alleles. We observed that expression of transgenic wild type PINCH-Flag significantly increased the frequency of hemizygous *flw* adult male escapers, and restored their fertility (Figure 1A, 1B). These crosses were done at 18°C because the frequency of adult escapers for any genotype was extremely low if reared at 25°C. A second, independent insertion line expressing wild type PINCH-Flag recapitulates the increased frequency of adult escapers (data not shown).

**Figure 2:**
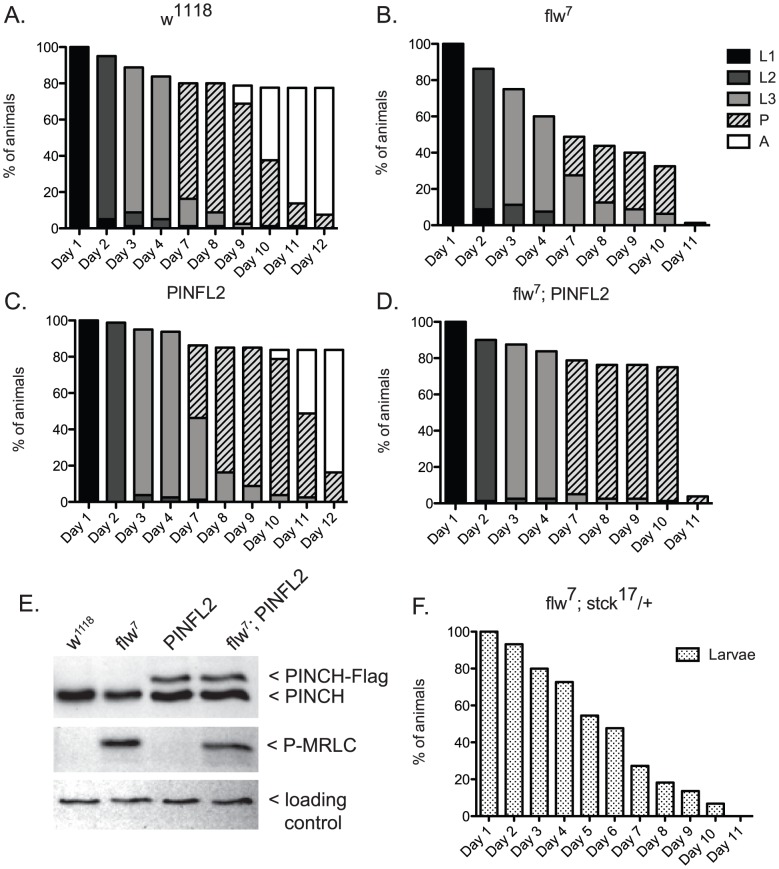
Expression of PINCH-Flag fully rescues the larval lethality of the *flw^7^* mutant independently of MRLC/Sqh dephosphorylation. A–D) For each genotype, graphs show the percentage of animals of the given developmental stage at the indicated time points. Pupae present at the final time point shown in A and C eventually eclosed as adults, whereas pupae in B and D subsequently arrested. E) Representative western blot of four-day-old L3s confirms that PINCH expression is elevated in the *PINFL2* and *flw^7^; PINFL2* larval lysates (transgenic PINCH is 44% of endogenous levels for both samples, by densitometric analysis) and that PINFL2 expression does not affect the phosphorylation state of MRLC/Sqh. The ribosomal protein RACK1 is used as a loading control. F) Survival of *flw^7^; stck^17^/+* larvae (n = 44) is plotted against time.

To more carefully characterize the spectrum of defects in *flw* mutants, we assessed the viability across development at 25°C of male hemizygotes selected from balanced stocks. Compared to a wild type *w^1118^* control, we found significant larval lethality—approximately 70% of *flw^7^* larvae failed to successfully pupariate, and of the 30% that do form pupae, essentially all arrest development prior to adult eclosion (Figure 2A, 2B). Expression of transgenic PINCH-Flag in a wild type background at approximately 40% of endogenous levels had no significant effect on progression to adulthood when compared to wild type animals (Figure 2A, 2C, 2E). However, this modest expression of PINCH-Flag fully rescued the larval lethality of the *flw^7^* mutant (Figure 2B, 2D, 2E). At 25°C (unlike the 18°C data in Figure 1), pupal lethality is not rescued by elevated expression of PINCH-Flag (Figure 2D), which may reflect a more stringent requirement for Flapwing phosphatase activity in the pupa to adult transition at higher temperature. The larval lethality of the *flw^6^* allele is similarly rescued by PINCH-Flag expression (Figure S1). Because of the similarity between *flw^6^* and *flw^7^* in 1) the number of adult escapers at 18°C, 2) the developmental profile at 25°C, and 3) the rescue of larval lethality by expression of transgenic PINCH, we focus on *flw^7^* in the remaining experiments.

We also tested whether a reduction in PINCH exacerbates *flw* lethality. Decreasing PINCH dosage using a heterozygous *stck^17^* mutation in the *flw^7^* background was challenging due to a high rate of balancer breakdown in the required crosses. However, the limited number of *flw^7^; stck^17^/+* animals that were isolated exhibited complete larval lethality (n = 44) (Figure 2F). These data confirm that reducing PINCH gene dosage indeed enhances *flw* lethality.

One way that increased PINCH-Flag expression could rescue *flw* larval lethality is through reversing protein hyper-phosphorylation that results from *flw* loss-of-function. Because the sole essential activity of Flapwing is the dephosphorylation of non-muscle myosin regulatory light chain (MRLC)/Sqh [11], we examined the phosphorylation state of MRLC/Sqh in larval lysates. As expected, phospho-MRLC was elevated significantly in *flw^7^* lysates as compared to wild type *w^1118^* controls (Figure 2E). However, statistical analysis of four independent replicate experiments showed that expression of PINCH-Flag, either in wild type flies or in the *flw* mutant background, had no effect on the phosphorylation state of MRLC (Figure 2E). These data indicate that regulation of MRLC phosphorylation state by PINCH is not the mechanism for the genetic suppression we observe between *flw* and PINCH.

We also examined the phosphorylation states of several other phospho-proteins that have been connected to PINCH: Akt [31], JNK [19], and ERK [32]. We found that phosphorylation of these proteins is not consistently elevated in larval lysates from *flw* mutants (data not shown), indicating either that Akt, JNK, and ERK are not actively signaling in these samples, are not substrates of Flapwing, or that other phosphatases compensate to appropriately dephosphorylate Akt, JNK and ERK in the *flw* mutant. While it is formally possible that PINCH could contribute to the phospho-regulation of non-essential Flapwing targets, it seems unlikely that phospho-regulation is a major contributor to the suppression of *flw* lethality observed upon elevated PINCH expression.

**Figure 3:**
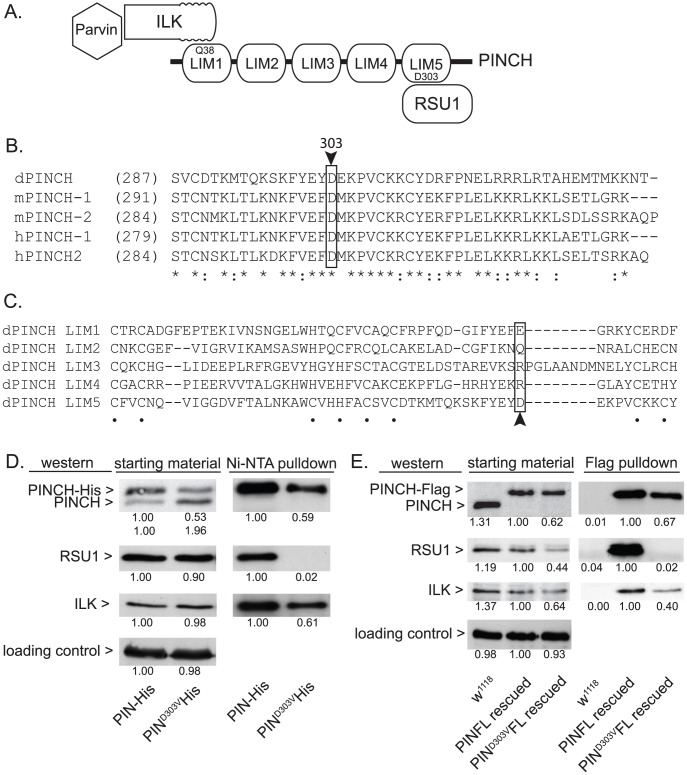
PINCH^D303V^ mutation disrupts binding to RSU1. A) Schematic of PINCH showing its 5 LIM domain structure. The N-terminal ankyrin repeat of ILK binds to LIM1 of PINCH via PINCH^Q38^. Parvin associates with PINCH indirectly via binding to the ILK pseudokinase domain. RSU1 binds to LIM5 of PINCH through PINCH^D303^. B) Sequence alignment of the C-terminus of fly, mouse and human PINCH sequences demonstrates the conservation of D303 (boxed with arrowhead). Asterisks denote invariant residues, and colons denote conserved residues. C) Sequence alignment using the 5 individual LIM domains of *Drosophila* PINCH shows that D303 (boxed with arrowhead) is a variable residue within LIM sequences. Dots denote the zinc binding residues. D) Ni-NTA pull-downs of wild type PINCH-His and PINCH^D303V^His expressed in S2 cells show a disruption of RSU1 binding only in the D303V mutant while ILK binding is preserved. E) Flag immunoprecipitations of adult fly lysates from PINCH null mutants rescued with either wild type PINCH-Flag or PINCH^D303V^Flag transgenes. *w^1118^* is used as a negative control that does not express Flag. ILK, RSU1 and a PINCH transgene are all expressed in the starting material. PINCH^D303V^Flag pulls down ILK but fails to co-precipitate RSU1. In both D and E, densitometric analyses were conducted to compare levels of protein between samples and quantification is provided below each blot. α-tubulin was used as a loading control and values for PINCH, RSU1 and ILK were adjusted to account for variation in loading.

In order to molecularly dissect the mechanism by which PINCH is suppressing *flw* larval lethality, we employed several mutants of PINCH that disrupt binding of its known partners, Integrin-linked kinase (ILK) and Ras Suppressor 1 (RSU1) (Figure 3A). One of these mutants, PINCH^Q38A^, has previously been shown to disrupt binding of ILK to LIM1 of PINCH (Figure 3A) [30], [33], [34], allowing us to test whether ILK binding and PINCH-ILK complexes are participating in the suppression of *flw* lethality. We were also interested in testing the contribution of RSU1 binding to LIM5 of PINCH (Figure 3A), and the role PINCH-RSU1 complexes. Mutations in PINCH that disrupt RSU1 binding have not yet been described. Therefore, we designed a modified yeast two-hybrid screen to identify point mutations in LIM5 of PINCH that specifically disrupt binding to RSU1 while otherwise preserving the structure and function of PINCH. We identified PINCH^D303V^ as a strong candidate. Among PINCH LIM5 sequences, D303 is highly evolutionarily conserved (Figure 3B), suggesting its importance for the proper functioning of PINCH. However, D303 is a variable residue within the general LIM consensus sequence (Figure 3C) and can therefore be altered without destroying overall LIM structure. The disruption of RSU1 binding was tested by inserting the D303V mutation into full length, Histidine-tagged PINCH expressed in *Drosophila* S2 cells. In the cell lysates used for purification, we routinely observe that PINCH^D303V^-His is present at reduced levels as compared to wild type PINCH-His. PINCH that is not bound to RSU1 has previously been shown to be less stable [19], [35]. Even so, Ni-NTA purification of the PINCH-His complexes confirms that PINCH^D303V^His, in contrast to a wild type PINCH-His control, clearly does not co-purify with RSU1 (Figure 3D). Moreover, PINCH^D303V^His retains the ability to bind to ILK (Figure 3D), indicating that folding and stability of the mutant PINCH^D303V^ have not been completely destroyed.

To further confirm that the PINCH^D303V^ mutation disrupts RSU1 binding, we next expressed PINCH^D303V^Flag in flies using the native PINCH promoter. In a wild type genetic background where the transgene is expressed at low levels, we did not observe any obvious dominant effects (data not shown). When this transgene was introduced into the *stck^17^/stck^18^* PINCH null background, viable rescued adults were produced at 90% of the expected frequency (n = 209). In soluble extracts made from these rescued adults, PINCH^D303V^Flag is routinely present at levels that are reduced compared to animals expressing either endogenous PINCH or rescued with the wild type PINCH-Flag transgene. In an effort to understand why PINCH^D303V^ is present at lower levels, we performed semi-quantitative RT-PCR on mRNA isolated from adult flies lacking endogenous PINCH and rescued with either wild type, Q38A, or D303V versions of the PINCH transgene. These mRNAs differ only in the sequence of a single codon. We find that levels of each transgenic message are equal (Figure S2), confirming that differences in the levels of PINCH protein occur post-transcriptionally. Additionally, levels of protein for RSU1 and ILK were reduced in the PINCH^D303V^Flag animals (Figure 3E), likely reflecting a well-characterized destabilization of the proteins in this complex when intact PINCH complexes are disrupted [19], [22], [28]–[30]. While PINCH^D303V^Flag rescued animals are viable as adults, wing blisters are frequent, the wings are often observed in a “held up” posture (similar, for example, to mutations in the Troponin genes *wupA* and *wupB* [12]), and the rescued adults are flightless. These phenotypes are comparable in severity to those of the viable RSU1 null mutant [19], and demonstrate the importance of the PINCH-RSU1 interaction. Flag pull-downs from rescued adult lysates confirm that both ILK and RSU1 strongly associate with wild type PINCH-Flag. Similar to the cell culture experiments, PINCH^D303V^Flag clearly retains the ability to bind ILK, but no RSU1 is detected in the PINCH^D303V^Flag pull-downs (Figure 3E). This confirms that the PINCH^D303V^ mutation effectively disrupts the binding of RSU1.

**Figure 4:**
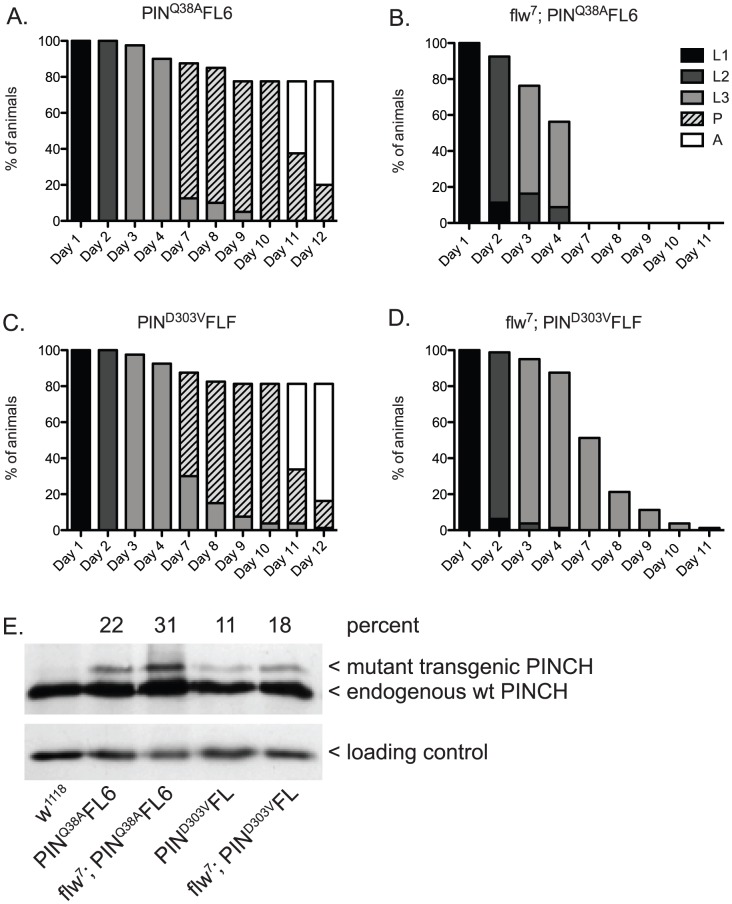
PINCH associates with ILK and RSU1 in order to suppress the larval lethality of *flw^7^* mutants. Survival curves show normal developmental progression upon expression of transgenic PINCH^Q38A^Flag (A) or PINCH^D303V^Flag (C) alone. Pupae present at day 12 subsequently progress to adulthood. Upon introduction of the mutant PINCH transgenes into a *flw^7^* background (B,D), arrest during the larval stages is observed. E) Western blot plus densitometric analysis for PINCH shows the relative level of expression of the mutant Q38A and D303V transgenes as a percentage of endogenous PINCH. The ribosomal protein RACK1 was used as a loading control.

Similar to the experiment (Figure 2) in which we suppressed larval lethality by expressing wild type PINCH-Flag in the *flw^7^* background, we expressed low levels of transgenic PINCH^Q38A^Flag along with endogenous wild type PINCH (Figure 4B, 4E). Unlike wild type PINCH-Flag expression, expression of the PINCH^Q38A^Flag mutant in the *flw^7^* background does not allow for suppression of larval lethality (Figure 4B compare to Figure 2B). This occurs despite viability of the PINCH^Q38A^ transgene expressed in a wild type background that is comparable to the wild type *w^1118^* control (Figure 4A compare to Figure 2A), and the full rescue of *stck* null mutants by PINCH^Q38A^ [35]. These data indicate that PINCH-ILK complexes participate in the suppression of *flw* larval lethality. Expression of PINCH^Q38A^Flag, which differs from the wild type transgenic protein in only a single amino acid and in its ability to bind ILK, has no capacity to promote pupariation in the *flw* mutant.

Next, we tested for suppression of *flw* larval lethality by co-expressing transgenic PINCH^D303V^Flag along with endogenous wild type PINCH in the *flw^7^* background (Figure 4D, 4E). Like PINCH^Q38A^Flag, low levels of expression of PINCH^D303V^Flag do not suppress *flw* larval lethality. Despite the pupal and adult viability of PINCH^D303V^Flag expressed in a wild type background (Figure 4C, 4E), pupariation was never observed in the *flw^7^; PINCH^D303V^Flag* animals (Figure 4D). Taken together, this suggests that intact ILK-PINCH-RSU1 complexes are necessary for the rescue observed in the *flw* mutant upon expression of transgenic PINCH. It is interesting to note that not only do the mutant PINCH transgenes fail to rescue *flw^7^* lethality, they in fact exacerbate the larval lethality, eliminating pupariation entirely. We comment on possible mechanisms for this enhancement as well as its implications below.

How does increased expression of PINCH and formation of functional PINCH complexes allow for improved survival of *flw* mutant larvae? One possibility is that in order to cope with the downstream effects of compromised protein dephosphorylation and a constitutively contracted actomyosin cytoskeleton, increased levels of PINCH and its associated binding partners strengthen integrin-dependent adhesions. Mutant analyses of PINCH and ILK in the fly indicate that these proteins have a role in maintaining embryonic muscle attachment [23], [24], and both *Drosophila* and *C. elegans* RNAi experiments confirm a role for these proteins in adult muscle maintenance [15], [36], [37]. If moderately elevated expression of PINCH leads to stronger adhesions, this may allow the *flw* mutants to better withstand the elevated mechanical stress they experience. To test this idea, we performed a series of morphological and functional assays to characterize *flw* mutants.

**Figure 5:**
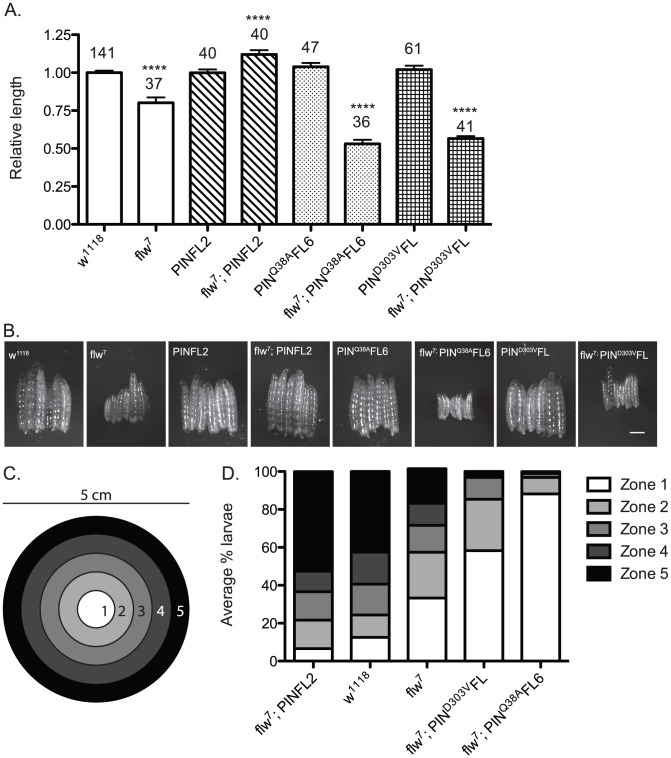
Moderately elevated expression of wild-type PINCH rescues the size and motility defects of *flw^7^* mutants. A) The lengths of four-day-old larvae were measured, normalizing to the mean of a parallel *w^1118^* control. Mean±SEM for each genotype is shown, with the number of animals measured (n) shown above the bar on the graph. **** indicates p<0.0001 compared to the *w^1118^* control. B) Representative phase contrast images of six L3 larvae of the indicated genotypes. Scale bar = 1 mm. C) The larval motility assay uses a 5 cm grape agar plate divided into zones as shown. D) The average percentage of larvae located in each zone at the end of the motility assay is plotted for the indicated genotypes, arranged in order of decreasing motility.

In addition to reduced viability, *flw^7^* mutant larvae have decreased and more variable size as compared to a *w^1118^* wild type control (Figure 5A, 5B). The small size of the *flw* larvae may result from either developmental delay or arrest, or be a secondary effect of feeding deficits due to poor muscle function. Expression of PINCH-Flag alone does not affect larval size (Figure 5A, 5B). However, elevated expression of PINCH-Flag in the *flw* mutant produces animals that are significantly larger than even the *w^1118^* wild type control (Figure 5A, 5B). Likewise, expression of either PINCH^Q38A^Flag or PINCH^D303V^Flag alone has no effect on larval size (Figure 5A, 5B). However, expression of PINCH^Q38A^Flag or PINCH^D303V^Flag in the *flw* background fails to restore normal larval size and yields animals that are significantly smaller than the *flw* mutant alone (Figure 5A, 5B).

We next analyzed these same genotypes of animals in a motility assay that measures spontaneous larval crawling through five zones on a grape juice agar plate (Figure 5C). Wild type *w^1118^* larvae, as well as matched samples expressing the PINCH-Flag, PINCH^Q38A^Flag or PINCH^D303V^Flag transgenes, exhibit robust and comparable levels of larval motility (Figure 5D and Figure S3). *flw* larvae exhibit decreased motility, which is rescued by PINCH-Flag expression (Figure 5D). In fact, the *flw^7^; PINFL2* animals are significantly more motile than even the wild type controls, perhaps due to their larger size. Expression of either PINCH^D303V^Flag or PINCH^Q38A^Flag respectively in the *flw^7^* mutant fails to rescue larval motility and increasingly exacerbates the motility defects of *flw^7^* animals (Figure 5D). These data show that 1) moderately elevated expression of PINCH rescues muscle function required for larval crawling, and 2) mutant PINCH transgenes that cannot form functional ILK-PINCH-RSU1 complexes fail to rescue larval size and motility, as well as overall viability.

**Figure 6:**
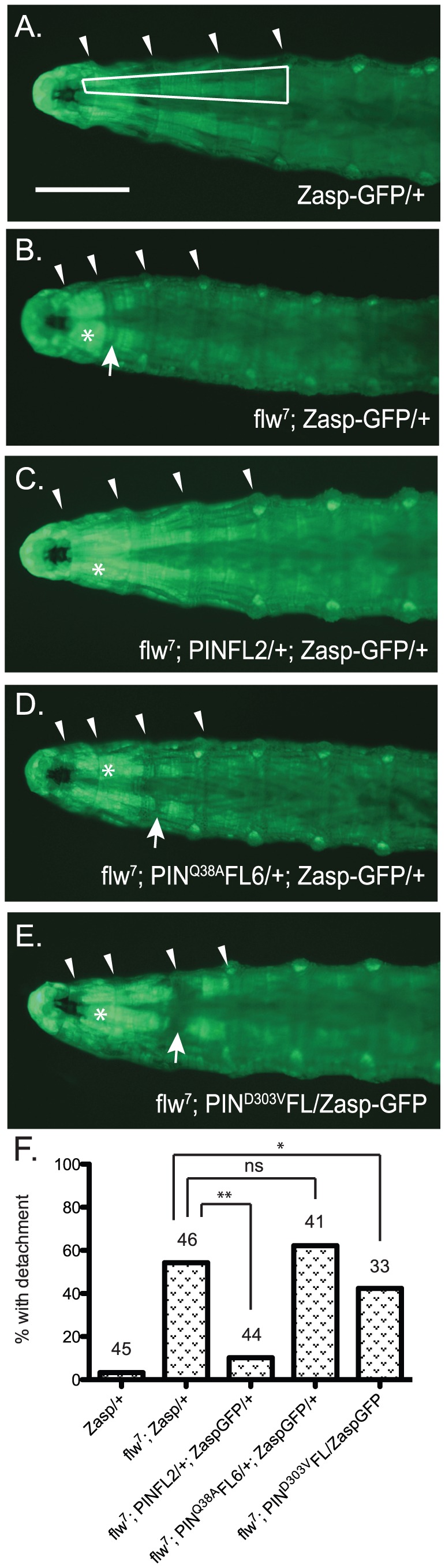
Moderately elevated expression of PINCH rescues muscle detachment defects of the *flw^7^* mutant. A–E) Representative images of four-day-old larvae of the indicated genotypes are shown. Ventral view allows the VIS muscles to be rapidly scored on a dissecting microscope. One of the medial pair of VIS muscles is boxed in A. White arrowheads show the relevant segment boundaries (T1,T2,T3). Areas of hypercontraction are indicated with an asterisk. Detachments are characterized by a gap in the GFP signal (arrow) Scale bar = 1 mm. F) The graph shows the percent of total animals in which the VIS muscles exhibit detachment. Number of animals examined of each genotype, pooled from triplicate experiments, is shown above the bars. ** indicates p<0.005, * indicates p<0.05, and ns indicates p>0.05.

Assessing structural integrity of the muscle in the *flw* larvae presents several technical challenges: antibody reagents cannot penetrate the larval cuticle of intact animals, and the physical manipulations required for dissection introduce structural defects not present in the intact larvae, particularly because the muscle integrity in these animals is already compromised. To circumvent these issues, we crossed the *flw^7^* mutant with a *Zasp66^ZCL0633^* P-element insertion line, which contains a GFP coding sequence inserted into the genomic locus for the muscle-specific PDZ protein, Zasp66 [38]. Zasp-GFP localizes normally to muscle Z-lines and has been extensively used to visualize muscle structure without the need for dissection in a variety of genetic backgrounds [39]. Although there is failure in pupation later in development, the insertion of GFP into the *Zasp66* locus does not significantly impact the viability or motility of the *flw^7^* mutant larvae at the four-day time point we used to analyze muscle integrity (Figure S4). *flw* mutants are reported to exhibit larval muscle detachment [10], and indeed in the *flw^7^; Zasp-GFP* larvae we observe frequent detachment, particularly in the Ventral Intersegmental (VIS) muscles. Because of the ease with which the VIS muscles can be scored for detachment, we have used this group of muscles as a read-out for muscle integrity. Zasp-GFP controls show very little detachment of the VIS muscles (Figure 6A, 6F). Additionally, expression of a single copy of the PINCH-Flag, PINCH^Q38A^Flag or PINCH^D303V^Flag transgenes in the Zasp-GFP background does not alter the normal attachment of the VIS muscles (Figure S5). However, *flw* mutants show frequent VIS muscle detachment (Figure 6B, 6F) that is partially rescued by expression of a single copy of the wild-type PINCH-Flag transgene (Figure 6C, 6F). Notably, hypercontraction, evidenced by areas of saturated Zasp-GFP signal (Figure 6B–6E, asterisks), is still frequently observed in the *flw^7^; PINFL2/+; Zasp-GFP/+* animals (Figure 6C). This is consistent with the idea that PINCH is not eliminating hypercontraction, but rather stabilizes integrin adhesions to prevent muscle detachment and subsequent larval lethality in the *flw* mutants. In contrast to the robust rescue of detachment observed upon transgenic expression of wild type PINCH-Flag in the *flw^7^* mutant, expression of PINCH^Q38A^Flag does not rescue detachment, and the PINCH^D303V^Flag transgene does not rescue muscle detachment to the same extent (Figure 6D–6F). The frequency of detachment upon transgenic PINCH expression mirrors the level of lethality observed in four-day-old larvae of the corresponding genotype (Figure 2, Figure 4, Figure S5). These data suggest that an increase in the number of intact PINCH-ILK-RSU1 complexes enables retention of muscle attachments in the *flw* mutant background.

**Figure 7:**
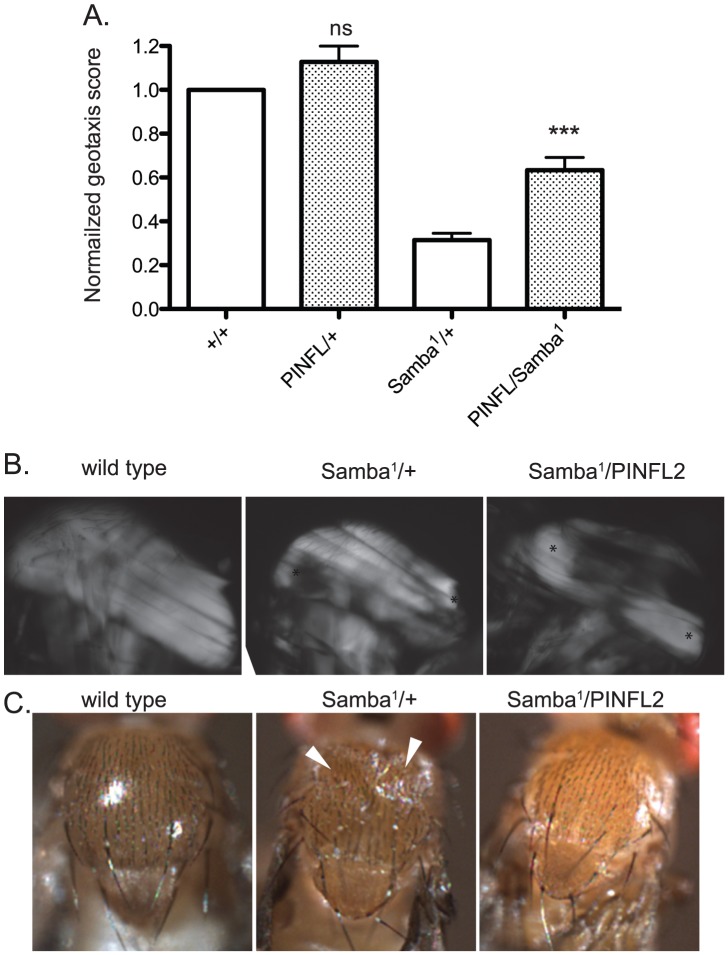
Increased expression of PINCH-Flag rescues geotaxis defects and thoracic indentation, but does not reverse hypercontraction of the IFM in the *Samba^1^* mutant. A) For each genotype, a normalized geotaxis score from >5 independent assays is plotted as the mean±SEM. *** indicates p<0.001 and ns indicates p>0.05 when compared to the corresponding control data. B) Representative polarized light images of thoraces show that the IFM in the *Samba^1^* mutant exhibits hypercontraction that is not rescued upon expression of PINCH-Flag. Asterisks indicate areas of hypercontraction within the structure of the IFM. C) A dorsal view shows that the thoracic indentations prevalent in *Samba1/+* adults are rescued upon expression of transgenic PINCH-Flag. White arrowheads indicate the areas of indentation.

If moderately elevated expression of PINCH is rescuing *flw* phenotypes by stabilizing integrin-based adhesions, increased expression of PINCH might also be expected to rescue the phenotypes of other hypercontraction mutants. To test this, we employed a dominant hypercontraction mutant in muscle Myosin Heavy Chain called *Mhc^Samba1^* [14]. The molecular lesion of *Mhc^Samba1^* is in the ATP binding/hydrolysis domain, producing muscle hypercontraction by a direct molecular mechanism distinct from the *flw* mutants. The muscle defects of *Mhc^Samba1^* are apparent in heterozygous adults in a geotaxis assay in which animals are induced to climb [15], [40]. *Mhc^Samba1^* heterozygous mutant adults exhibit poor climbing ability that is significantly improved upon expression of transgenic PINCH-Flag (Figure 7A). These data support the idea that increased expression of PINCH generally stabilizes muscle attachments where hypercontraction is present.

To further characterize the rescue of *Samba^1^* mutants by transgenic PINCH-Flag expression, we examined indirect flight muscle (IFM). IFM hypercontraction can be seen by polarized light microscopy in the *Samba^1^* mutant ([9] and Figure 7B), and renders these animals flightless [9]. Consistent with the idea that elevated PINCH expression is stabilizing integrin attachments rather than reversing hypercontraction, the IFM of *Samba^1^/PINCH-Flag* adults does not resemble wild type IFM, but continues to exhibit hypercontraction (Figure 7B), and these flies remain flightless (data not shown). As a consequence of IFM hypercontraction, a portion of *Samba^1^/+* animals (39%, n = 70) exhibit visible external thoracic indentations in freshly eclosed adults ([9] and Figure 7C). Although expression of PINCH-Flag in the *Samba^1^* heterozygotes does not rescue IFM hypercontraction, it dramatically improves the thoracic indentation phenotype (2% indented, n = 62) (Figure 7C).

**Figure 8:**
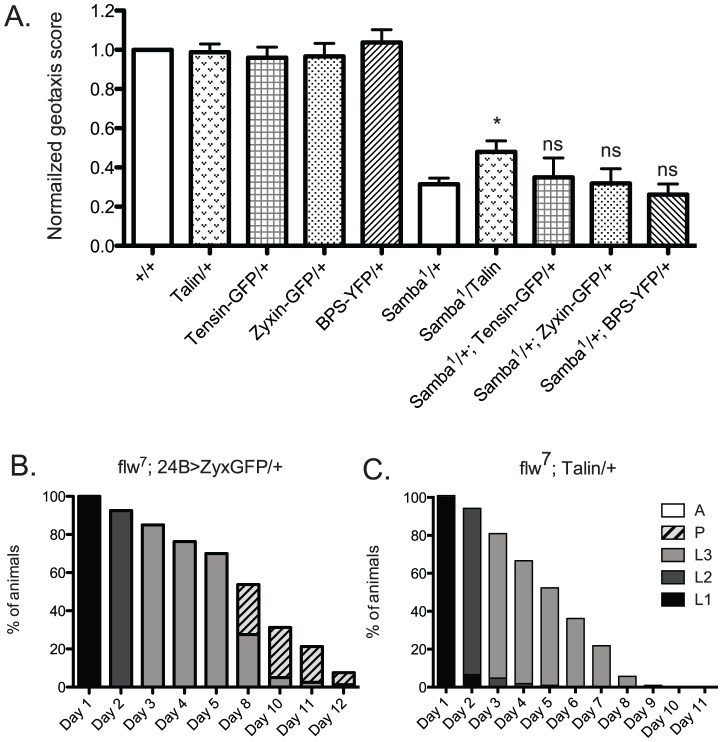
Increased expression of a panel of IAC proteins has little effect on hypercontraction mutants. A) For each genotype, a normalized geotaxis score from >5 independent assays is plotted as the mean±SEM. * indicates p<0.05, and ns indicates p>0.05 when compared to the *Samba^1^/+* data. B) Survival data for *flw^7^; 24B>Zyxin-GFP/+* is not significantly different than *flw^7^* alone (compare to Figure 2B). Pupae present at day 12 do not progress to adulthood. C) Survival data for *flw^7^; Ubi-Talin/+* shows that larval lethality is increased compared to *flw^7^* alone (see Figure 2B).

We next wanted to determine whether increased expression of other integrin adhesive complex (IAC) components might be protective under conditions of hypercontraction, or whether PINCH is unique in this regard. To test this, beginning with the geotaxis assay in the *Samba^1^/+* mutant background, we employed a panel of IAC transgenic proteins to assess suppression of hypercontraction-induced climbing defects. This panel included ILK-GFP and Tensin-GFP controlled by their native promoters, FAK-GFP and Zyxin-GFP expressed using the Gal4-UAS system and the muscle-specific 24B driver, and Ubi-βPS-Integrin-YFP and Ubi-Talin expressed from the *ubiquitin* promoter. From among this panel, ILK-GFP was not overexpressed and FAK-GFP expression was lethal prior to adulthood [41], so these proteins were not tested in the geotaxis assay. Western analyses demonstrated increased expression of Zyxin-GFP, βPS-Integrin-YFP, and Talin in adult fly lysates (data not shown). We could not confirm expression of Tensin-GFP because of lack of antibody reagents, but this transgene has previously been shown to express, as it rescues null mutations in the Tensin gene *blistery* [42]. The presence of the Tensin-GFP, Zyxin-GFP, and βPS-Integrin-YFP transgenes had no significant effect on the climbing ability of *Samba^1^* mutants (Figure 8A). Of the additional IAC proteins tested, only transgenic Talin expression resulted in a modest improvement of the geotaxis score for the *Mhc^Samba1^* mutant (Figure 8A). Rescue of IFM hypercontraction and thoracic indentation was not observed with expression of transgenic Talin (data not shown). This suggests that if increased expression of Talin does strengthen integrin adhesions, it is unlikely to do so in the same manner as PINCH.

In efforts to bolster the *Samba^1^* geotaxis data, we further analyzed two of the panel of IAC proteins in the *flw* genetic background: Zyxin and Talin. We first analyzed the effect of Gal4-UAS expression of Zyxin-GFP from the muscle-specific 24B driver on the developmental profile of *flw^7^*. Animals expressing 24B>Zyxin-GFP alone show normal developmental progression (data not shown). As predicted from the lack of rescue of *Samba^1^* geotaxis, we see no significant difference in either the rate of larval or pupal lethality upon expression of Zyxin-GFP in the *flw^7^* mutants (Figure 8B, compare to Figure 2B). Next, we showed that upon expression of Ubi-Talin in the *flw^7^* background, we did not observe any rescue of larval lethality (Figure 8C). In fact, pupariation was blocked entirely in *flw^7^; Talin/+* animals. Because transgenic Talin expression does not uniformly rescue both of the hypercontraction mutants tested, the biological significance of the marginal rescue by Talin in the *Samba^1^/+* geotaxis assay is unclear. Moreover, these data suggest a high degree of specificity in the rescue of multiple hypercontraction phenotypes by PINCH expression.

## Discussion

In this report, we tested whether elevated expression of the IAC protein PINCH, as a means to strengthen integrin-based adhesions, can alleviate the phenotypes associated with muscle hypercontraction. We show that moderately elevated expression of PINCH dramatically rescues the lethality as well as the size, motility and muscle attachment defects associated with the myosin phosphatase hypercontraction mutant, *flw*. The interaction of PINCH with its binding partners ILK and RSU1 is required for rescue. Elevated PINCH expression neither alters the phosphorylation state of the essential Flapwing substrate MRLC, nor alleviates the hypercontraction observed in the larval VIS muscles, but instead may rescue through the stabilization of integrin-mediated adhesions. This idea is further supported by the partial rescue of the climbing deficits of an independent hypercontraction mutant in muscle myosin heavy chain, *Mhc^Samba1^*, as well as the rescue of its thoracic indentation phenotype, without reversal of the hypercontraction evident in the IFM.

Initial work on this project included testing for interactions between PINCH and PP1 phosphatase family members in *Drosophila*. Data in mammalian cells shows that PP1α binds to PINCH via a KFVEF sequence motif present in LIM5 of PINCH, and that binding inhibits the phosphatase activity of PP1α [31]. We tested extensively for direct binding between Flapwing and PINCH in *Drosophila* using a variety of biochemical approaches, but were unable to demonstrate a physical interaction (data not shown). Moreover, if PINCH-PP1 binding and inhibition were evolutionarily conserved, we predicted that elevated expression of PINCH in *Drosophila* should exacerbate the defects of a *flw* hypomorph and lead to further hyper-phosphorylation of Flapwing targets like MRLC. We did not observe either of these things—rather, elevated expression of PINCH rescued *flw* phenotypes and had no effect on the phosphorylation state of the Flapwing target MRLC. There are several possible explanations for these discrepancies. First and least interesting, a PINCH-PP1 direct binding interaction may simply not be conserved in *Drosophila*—an idea we cannot disprove with negative biochemical data. Second, Flapwing is a PP1β family member rather than PP1α. While there is a high degree of sequence similarity between the two branches of the PP1 family, the distinguishing residues might play a key role in specifying PINCH binding. Our experiments do not address whether PINCH directly binds and inhibits PP1α isoforms in *Drosophila*, but rather show that PINCH is having a distinct and separate effect under conditions of PP1β loss-of-function via the stabilization of integrin-based adhesions. Third and most intriguing, the role of RSU1 in the PINCH-PP1α interaction is not yet clear. Both RSU1 and PP1α bind to LIM5 of mammalian PINCH. We have shown that PINCH^D303^ is a key residue for the binding of RSU1. Notably, the mammalian PINCH^KFVEF^ motif that is responsible for PP1α binding [31] spans the immediately adjacent residues corresponding to 298–302 in the fly protein, which are moderately well conserved (KFYEY). As such, the binding interface on PINCH that participates in PP1α binding is likely to be overlapping with the RSU1 binding interface. It remains to be determined whether PINCH can bind both RSU1 and PP1α simultaneously, whether binding of PINCH to RSU1 and PP1α is mutually exclusive, or whether RSU1 rather than PINCH directly binds PP1α. Mutational analyses on the KVFEF motif of mammalian PINCH disrupted the association with PP1α [31], but it is formally possible that the KFVEF mutant disrupts RSU1 binding, preventing RSU1-PP1α complexes from docking on PINCH. Further studies on the mammalian versions of these proteins will be necessary to distinguish between these possibilities.

Expression of the binding mutants, PINCH^Q38A^Flag and PINCH^D303V^Flag, resulted in strong phenotypes in the context of Flapwing loss-of-function, eliminating pupariation entirely. Of note, expression from the Ubi-Talin transgene or the presence of the Zasp66-GFP gene trap had the equivalent effect of failed *flw* pupariation. This suggests that in the *flw* background, developmental events required for pupariation are particularly prone to disruption by alterations in the IAC. This makes the dramatic rescue of *flw* pupariation upon expression of PINCH-Flag all the more remarkable.

Expression of PINCH^Q38A^Flag and PINCH^D303V^Flag produces no dominant effects in a wild type background. There appears to be plasticity in IAC formation/maintenance to tolerate the presence of a small fraction of incomplete mutant PINCH complexes [35]. However, expression of the mutant PINCH transgenes creates a background that is sensitized to further perturbations. Upon *flw* loss-of-function, the presence of incomplete mutant PINCH complexes has severe and reproducible consequences on viability, size, and motility. There is a clear synergistic effect of combining *flw* loss-of-function and the corresponding alterations in protein phosphorylation, with expression from the mutant PINCH transgenes.

Our data suggest that PINCH^Q38A^Flag and PINCH^D303V^Flag participate in the formation of incomplete/unproductive PINCH complexes that may act dominantly in the *flw* background. This is consistent with numerous examples in cell culture in which overexpression of mutant forms of PINCH titrates away crucial binding partners to dominantly affect processes such as protein localization and cell spreading [34], [43]–[47]. The effect of mutant PINCH expression on the viability of *flw* animals is substantial, despite the relatively small amount of PINCH^Q38A^Flag and PINCH^D303V^Flag protein that is expressed (approximately 20–30% of endogenous PINCH). This may occur because of the mutual protein stabilization phenomenon exhibited by RSU1-PINCH-ILK-Parvin complexes [19], [22], [28]–[30]. It is plausible that incomplete complexes containing mutant PINCH may be destabilized and more rapidly turned over, resulting in insufficient numbers of intact complexes to maintain optimal adhesive function in the *flw* background. Indeed, in *C. elegans* muscle, incomplete integrin adhesive complexes have been shown to accelerate the rate of IAC protein degradation to disrupt myofibrillar and mitochondrial morphology [36], [37].

PINCH-Flag expression rescues the phenotypes of three different hypercontraction mutations: *flw^6^* and *flw^7^* in non-muscle myosin phosphatase, and *Mhc^Samba1^* in muscle myosin heavy chain. This strongly supports the idea that elevated PINCH expression strengthens integrin-based adhesion, and more robust adhesion can mitigate hypercontraction-induced muscle damage. Transgenic expression of additional IAC components from readily available stocks was first tested by geotaxis in the *Mhc^Samba1^* mutant because of the ease of generating *Samba^1^* heterozygotes expressing transgenic IAC proteins. The results from this panel of transgenes were mixed. Some IAC proteins did not readily overexpress (ILK-GFP), suggesting that tight regulation induces more rapid turnover upon increased gene expression. Overexpression of another IAC protein was lethal (24B>FAK-GFP) and it therefore could not be tested in the rescue of adult *Samba* mutant phenotypes. Increased expression of several additional IAC proteins had no effect (24B>Zyxin-GFP, Tensin-GFP, Ubi-βPS-Integrin-YFP). Lack of rescue could result if increased expression does not serve to strengthen adhesions because that component is not limiting for IAC formation or function. Alternatively, if the IAC transgene is not adequately expressed in the appropriate tissues (e.g.: both muscle and tendon cells into which they anchor), the transgenic protein will be unable to effectively strengthen adhesion. The 3kb PINCH promoter employed appears to have a useful expression pattern in this regard. It remains to be seen whether other promoters and other IAC genes can direct expression that will strengthen adhesion to the same degree. It will be interesting in future studies to delineate which IAC proteins are limiting at muscle attachments as well as other adhesion sites, as better techniques to determine relative expression levels and stoichiometry are developed. Interestingly, Talin and Paxillin expression was upregulated in the microarray studies of the *Samba* mutants [14], suggesting that upregulation of select IAC proteins is a compensatory response to hypercontraction that is utilized *in vivo*. With elevated expression of PINCH-Flag, partial rescue of the *Samba* and flw defects was observed. This suggests that PINCH might be a limiting component of IACs, and increasing PINCH expression might serve to strengthen existing complexes and/or allow the assembly of additional IACs. Thus, strategic expression of PINCH, and perhaps other IAC proteins as well, may serve to preserve or protect muscle structure and function and may have therapeutic potential in myopathies that exhibit hypercontraction.

## Methods

### Drosophila Stocks

*flw^6^*, *flw^7^* (also known as *flw^G0172^*), *P[GawB]how^24B^*, and *Zasp66^ZCL0663^* were obtained from the Bloomington Stock Center. *Mhc^Samba1^* [9], *stck^17^* and *stck^18^* [23] have been previously described. Transgenic stocks include PINCH-Flag and PINCH^Q38A^Flag [30], UAS-Zyxin-GFP [48], ILK-GFP [24], UAS-FAK-GFP [41], Ubi-βPS-Integrin-YFP [49], Tensin-GFP [42], and Ubi-Talin [50].

### Measuring Frequency of Adult Escapers

Crosses between *w; PINFL2* and both *flw^6^/FM7i* and *flw^7^/FM7i* were set at 18°C and progeny counted. The percent escape was calculated as 100×[*flw* males/(total number of female progeny/2)]. Escaper male hemizygotes were tested for fertility by crossing to *w^1118^* virgin females.

### Survival Analyses

Stocks of the indicated genotypes were placed in cages and allowed to lay for approximately 6 hours on yeasted grape agar plates at 25°C. Except where noted, >80 animals of the appropriate genotype were selected from stocks as early L1s (24–30 hours) and placed on fresh grape agar plates. For stocks that are *FM7i* balanced, *flw* hemizygous males were selected by sorting against the GFP expressed from the *FM7i* balancer chromosome. A cross between *flw^7^/FM7i* and *FM7i/Y; stck^17^/TM3, twi>GFP* was used to generate *flw^7^; stck^17^/+* animals, by selecting against the GFP expressed on both balancer chromosomes. On subsequent days, the number of viable animals at each stage of development was counted and live animals were moved to a fresh plate. Putative larval stages were determined by size. In Figure 2F, the small number of animals in each cohort precluded this determination, and total larvae were counted without respect to putative stage. Any pupae present continued to be counted until the final time point presented in the graphs, when they were scored for viability by the presence of visible and healthy looking adult structures with no signs of necrosis or dehydration. All viable pupae were kept an additional 7 days to determine if they eventually eclosed as adults. A wild type *w^1118^* control was included to determine baseline lethality arising from experimental manipulation of the samples. Graphical analyses were done using GraphPad Prism.

For each analysis that involves a PINCH transgene, one of at least two independent insertion lines that were analyzed is shown, to confirm that the results were not dependent upon the locus of the transgene insertion.

### Western Analyses

Four-day-old L3 larvae were homogenized in RIPA buffer with phosphatase inhibitors and normalized by protein content (BioRad DC protein assay). Antibodies employed were anti-PINCH [23], anti-RSU1 [19], anti-ILK (BD #611802), anti-P-MRLC/Sqh (Cell Signaling), and anti-RACK1 [51] or anti-α-tubulin 12G10 (DSHB) as a loading control. All western analyses were performed at least three times and representative blots are shown. Densitometric analyses of western blots were performed using Image J.

### Modified Yeast Two-Hybrid Screen

In prior experiments that mapped the site of RSU1 interaction to LIM5 of PINCH, we constructed a LIM5 bait and RSU1 prey that activated an ADE2 reporter in a yeast two-hybrid system [19], [52]. Employing a low-fidelity polymerase, we amplified the LIM5 region of PINCH and sub-cloned it into the bait vector to create a randomly mutagenized LIM5 bait library. The RSU1-binding function of LIM5 is unaffected in most library clones, which display ADE2 reporter activity. In a fraction of the colonies, the LIM5-RSU1 interaction is disrupted. On non-selective plates, lack of ADE2 reporter activity allows a pink-colored precursor in the adenine biosynthetic pathway to accumulate. We analyzed pink colonies resulting from the co-transformation of the mutagenized PINCH LIM5 bait library and wild type RSU1 prey. Total DNA was isolated, and LIM5-encoding DNA was PCR amplified and sequenced. Frame shifts, truncations, or point mutations in the zinc ligands were not considered further. Sequence alignments were done using Clustal X.

### Plasmid Construction, Ni-NTA Pull-Downs, and Flag Immunoprecipitations

PCR mutagenesis of a previously described pMT-PINCH^wt^-His construct [19] was used to introduce an Aspartate to Valine mutation at position 303 in the dPINCHa cDNA. pMT-PINCH^wt^His and pMT-PINCH^D303V^His were stably transfected into S2 cells using standard methods and expression induced by the addition of CuSO_4_. S2 cell lysates were prepared in lysis buffer (50 mM Tris-HCl pH 7.9, 150 mM NaCl, 0.1% Triton-X 100) plus protease inhibitors, and were incubated with Ni-NTA agarose (Qiagen), clarified by centrifugation, washed with lysis buffer, then boiled in 2× Laemmli sample buffer prior to western blotting.

Transgenic flies carrying PINCH^D303V^Flag were generated by PCR mutagenesis of a previously described pCasper construct containing genomic PINCH^wt^-Flag [30]. This DNA construct was injected into embryos for p-element transposition by Genetic Services Inc. (Cambridge, MA). Transgenic flies were then crossed into a PINCH null background (*stck^17^/stck^18^*). Adult fly lysates were prepared in lysis buffer plus protease inhibitors, clarified by centrifugation and 0.45 µm filtration, and were incubated with anti-Flag M2 agarose (Sigma), washed, and boiled in 2× Laemmli sample buffer for western blotting.

### RT–PCR

Twelve adult flies per sample were lysed in 350 µl RLT Buffer using Qiashredder columns (Qiagen), and RNA was extracted using RNEasy Mini Kits (Qiagen) according to the manufacturer's recommendations. RT-PCR was conducted with 50 ng RNA per reaction using the Access RT-PCR System (Promega), with the following primers: PINCH-Flag (Forward: 5′GCACTGGCATGTGGAACATT3′, reverse: 5′ACTAGTCTACCTGTCATCGTC3′), GAPDH (Forward: 5′CAACTTCTGCGAAACGACAA3′, Reverse: 5′TGTCCTCCAGACCCTTGTTC3′).

### Larval Size and Motility Assays

Larvae of the given genotypes were sorted at 24–30 hr after egg lay and analyzed when they reached 4 days of age (3^rd^ instar). Prior to size measurements, larvae were heat fixed by rapidly submerging in boiling embryo wash (0.1% Triton X-100, 0.7% NaCl), and bright field images captured on an Olympus MVX10 dissecting microscope. Length measurements of individual larvae were normalized to the mean of a matched wild type *w^1118^* control. To measure larval motility, we adapted an existing protocol [53]. Eight four-day-old L3s of the desired genotype were placed onto a room temperature, 5 cm grape agar plate marked with zones of 5 concentric circles (Figure 5B). After acclimating for at least 5 minutes, all 8 animals were moved into zone 1, and the assay started. At 60 sec, the zonal location of all 8 animals was recorded. These measurements were performed in triplicate for each group of 8 larvae, and data for ≥4 independent sets (≥32 animals) were collected. The relative distribution of animals at the end of the motility assay was plotted in GraphPad Prism, using the mean percent of animals present in each zone.

### Ventral Intersegmental Muscle Morphology

Four-day-old L3s of the given genotypes were generated in a cross with *flw^7^/FM7i; Zasp-GFP*. Larvae were quickly heat fixed by rapidly submerging in boiling embryo wash (0.1% Triton X-100, 0.7% NaCl), then the Zasp66-GFP pattern in the Ventral Intersegmental (VIS) muscles was examined using an Olympus MVX10 GFP-dissecting microscope. Any animal in which the medial pair of VIS muscles does not correctly span body segments T1-T3 is scored as detached. Discontinuities in the Zasp-GFP signal are characteristic of breaks/detachments in the VIS muscles. Areas of hypercontraction are characterized by intense Zasp-GFP signal, as the Z-lines are more closely spaced. For each genotype, analyses were conducted in triplicate. Paired *t* tests were performed to determine statistically significant differences between genotypes.

### Geotaxis Assays

Assays were done according to a previously described protocol [15], [40]. At least 50 flies of each genotype were tested. In each assay, 10 adult flies (<48 hours post-eclosion) were transferred to an empty vial and lightly tapped to the bottom. The number of flies that climbed to a height of 7 cm within 8 seconds was recorded. For each vial, the measurement was repeated 4 times and averaged. To control for minor variations in assay conditions, all data points were normalized to a wild type control sample that was collected and analyzed in parallel (ie: the number of wild type flies that met climbing criterion was converted to a geotaxis score = 1.0, to which other genotypes were compared). At least five sets of normalized data were used to graph a geotaxis score±SEM. Paired *t* tests were performed to determine statistically significant differences between genotypes.

### Polarized Light Microscopy of Indirect Flight Muscle

IFM was analyzed as described previously [54], with several modifications. Briefly, adult flies of the indicated genotypes (n>20) were collected and dehydrated first in 100% ethanol, then in 100% isopropanol. Heads and abdomens were removed to speed equilibration. Thoraces were cleared >1 hour in xylenes, followed by >1 hour in BABB (1∶2 Benzyl Alcohol∶Benzyl Benzoate), then imaged in BABB using two external polarizing filters in conjunction with an Olympus MVX10 dissecting microscope. Thoracic indentations resulting from IFM hypercontraction were scored in >50 animals of each genotype upon visual inspection of freshly eclosed adults on a dissecting microscope.

## Supporting Information

Figure S1 Expression of the PINCH-Flag transgene rescues the larval lethality of the *flw^6^* mutant. A,B) For each genotype shown, graphs depict the percentage of animals of the given developmental stage at the indicated time points. Pupae present at day 11 do not progress to adulthood.
(EPS)

Figure S2 Differences in the levels of PINCH transgene expression are post-transcriptional. RT-PCR from adult fly lysates shows that mRNA levels transcribed from the wild type, Q38A and D303V versions of the PINCH transgene are identical. GAPDH is used as a loading control.
(EPS)

Figure S3 Expression of wild type or PINCH mutant transgenes alone does not significantly affect larval motility. The average percentage of larvae located in each zone at the end of the larval motility assay is plotted for the indicated genotypes.
(EPS)

Figure S4 Expression of Zasp-GFP in the *flw^7^* genetic background does not significantly alter viability or motility in four-day-old larvae. A) The graph shows the percentage of *flw^7^; Zasp-GFP* animals of the given developmental stage at the indicated time points. Expression of Zasp-GFP in the *flw^7^* mutant prevents pupariation, but at the time at which VIS muscle integrity is assessed (indicated by arrow), larval viability is not compromised (compare to Figure 2B), and B) motility is unaffected. The average percentage of larvae located in each zone at the end of the motility assay is plotted for the indicated genotypes.
(EPS)

Figure S5 Expression of wild type or mutant PINCH transgenes does not significantly affect VIS muscle attachment. The graph shows the percent of total animals in which the VIS muscles exhibit detachment. Number of animals examined of each genotype is shown above the bars.
(EPS)
